# Exploring the Influence of Cold Plasma on Epidermal Melanogenesis In Situ and In Vitro

**DOI:** 10.3390/ijms25105186

**Published:** 2024-05-10

**Authors:** Sybille Hasse, Marie-Christine Sommer, Sebastian Guenther, Christian Schulze, Sander Bekeschus, Thomas von Woedtke

**Affiliations:** 1 Leibniz Institute for Plasma Science and Technology e.V. (INP), a Member of the Leibniz Health Technologies Research Alliance, Felix-Hausdorff-Str. 2, 17489 Greifswald, Germany; marie-christine.sommer@inp-greifswald.de (M.-C.S.); sander.bekeschus@inp-greifswald.de (S.B.); woedtke@inp-greifswald.de (T.v.W.); 2 Institute of Pharmacy, Department Pharmaceutical Biology, Greifswald University, Friedrich-Ludwig-Jahn-Str. 17, 17489 Greifswald, Germany; sebastian.guenther@uni-greifswald.de (S.G.); christian.schulze@uni-greifswald.de (C.S.); 3 Institute for Hygiene and Environmental Medicine, Greifswald University Medical Centre, Walther-Rathenau-Str. 48, 17489 Greifswald, Germany

**Keywords:** epidermal melanocytes, melanogenesis, plasma jet, plasma medicine

## Abstract

Epidermal melanin synthesis determines an individual’s skin color. In humans, melanin is formed by melanocytes within the epidermis. The process of melanin synthesis strongly depends on a range of cellular factors, including the fine-tuned interplay with reactive oxygen species (ROS). In this context, a role of cold atmospheric plasma (CAP) on melanin synthesis was proposed due to its tunable ROS generation. Herein, the argon-driven plasma jet kINPen^®^ MED was employed, and its impact on melanin synthesis was evaluated by comparison with known stimulants such as the phosphodiesterase inhibitor IBMX and UV radiation. Different available model systems were employed, and the melanin content of both cultured human melanocytes (in vitro) and full-thickness human skin biopsies (in situ) were analyzed. A histochemical method detected melanin in skin tissue. Cellular melanin was measured by NIR autofluorescence using flow cytometry, and a highly sensitive HPLC-MS method was applied, which enabled the differentiation of eu- and pheomelanin by their degradation products. The melanin content in full-thickness human skin biopsies increased after repeated CAP exposure, while there were only minor effects in cultured melanocytes compared to UV radiation and IBMX treatment. Based on these findings, CAP does not appear to be a useful option for treating skin pigmentation disorders. On the other hand, the risk of hyperpigmentation as an adverse effect of CAP application for wound healing or other dermatological diseases seems to be neglectable.

## 1. Introduction

Melanin synthesis in human skin takes place in the epidermal melanocytes, which reside in the *stratum basale*. Melanocytes are also present in the hair bulb of the hair follicle, where they provide melanin to the growing hair. Epidermal melanocytes are neural crest-derived cells that have a dendritic shape. Specialized organelles called melanosomes contain melanin and are distributed to suprabasal keratinocytes within the epidermis. During this process, melanocytes can change their shape, dendricity and number of mature melanosomes depending on environmental challenges. The process of melanogenesis involves a complex, multi-stage process of different proteins and enzyme cascades, eventually resulting in the pigmentation of the skin and hair [1,2,3,4].

Melanin, especially eumelanin, has photoprotective properties. Under normal physiological conditions, UV-B radiation enhances melanogenesis and causes tanning of the exposed skin areas. This is a very important adaptive response, as it protects the skin from sun burn and decreases the risk of developing skin malignancies. However, excessive UV radiation can also have skin-damaging effects such as skin aging and DNA damage and eventually may lead to skin cancer. A distinct disruption or malfunction of skin pigmentation, for instance, in vitiligo, may have a great psycho-social impact on affected individuals [5,6].

Therefore, it is of great interest to identify potential tools to influence epidermal melanogenesis without any side effects. During the last two decades, the use of cold atmospheric plasma (CAP) for medical applications has been realized with a special focus on dermatology [7,8,9]. Although the potential application spectrum ranges from skin cancer to dentistry, the most advanced and investigated application is for wound healing. Several clinical studies and case series document its safe and effective use [10,11,12,13,14,15,16]. The advantages of CAP are the ability to inactivate a broad range of microorganisms, including multidrug-resistant strains and viruses. CAP enhances wound closure by accelerating angiogenesis, re-epithelialization and cell proliferation, and it modulates topical inflammation [16]. It therefore benefits healing in various types of wounds such as acute, chronic and diabetic wounds. On the other hand, the great diversity of CAP devices limits the consistency of treatment effects and comparability between applications [17].

CAP is sometimes referred to as the fourth state of matter. As such, it is generated by supplying (electrical) energy to a gas that becomes partially ionized. Consequently, it contains charged (electrons, ions) and neutral particles (molecules), as well as radiation components (UV, VIS, thermal) [18,19]. During the operation at ambient conditions, reactions with nitrogen and oxygen from surrounding air produce reactive oxygen species (ROS) and reactive nitrogen species (RNS), which are considered key components in CAP-mediated biological effects. During melanogenesis, many steps are finely tuned by ROS, including H_2_O_2_, i.e., the conversion of L-phenylalanine to L-tyrosine by phenylalanine hydroxylase (PAH) [20] and tyrosinase as key enzymes for melanin synthesis [21]. Considering this, it was highly intriguing to us to investigate a possible role of CAP in this process.

The aim of this study was to investigate the potential of CAP to stimulate melanogenesis and compare it to UV-B and 3-isobutyl-1-methylxanthine (IBMX) as well-known melanin promotors. To realize this aim, we used a variety of detection methods that included histochemistry, cytometry and HPLC/MS analysis.

## 2. Results

In order to assess the effects of CAP on the melanin content of melanocytes, two different test targets were employed. First, CAP treatment was applied ex vivo on the surface of full-thickness skin biopsies. Melanocytes reside in the basal layer and are tightly surrounded by keratinocytes that apically protect them by several layers of cells. In a second approach, cultured epidermal melanocytes were investigated, and CAP was applied indirectly as a plasma-treated cell culture medium. In contrast to the aforementioned test system, melanocyte cultures consist exclusively of melanocytes with no keratinocytes present. Both entities were explored using adequate detection approaches to evaluate the melanin content and its potential modulation by CAP.

### 2.1. Melanin Content in Human Skin Samples after CAP Exposure

**Figure 1:**
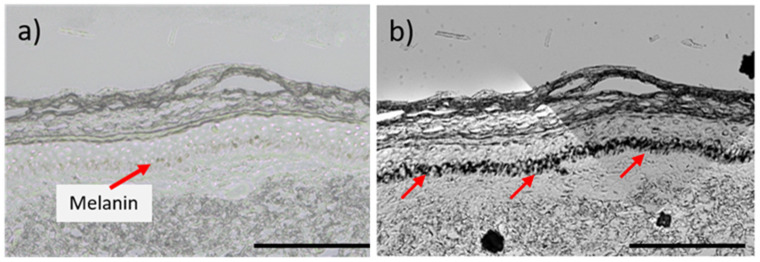
Representative images of cross sections of human skin: (**a**) Melanin-containing cells are barely visible at the basal layer of the epidermis, indicated by the red arrow. (**b**) Melanin granules appear dark black at the basal layer of the epidermis after Fontana–Masson staining (red arrows). Both skin samples are untreated. Scale bar 200 µm.

The impact of CAP on melanocytes residing inside full-thickness skin samples was evaluated by a histochemical method based on Fontana–Masson staining [22]. Skin biopsies were CAP treated through the stratum corneum, and cross-sectional skin sections were prepared after 24 h or 72 h, as indicated in Figure 1.

The Fontana–Masson staining formed black silver nitrate deposition along the basal and suprabasal layers. A representative image of the staining is shown in Figure 1b, with a strong black deposition where melanocytes reside. The melanin granules were quantified by image analysis.

**Figure 2:**
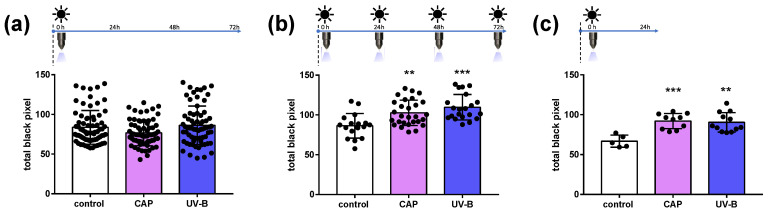
Quantitative image analysis of silver nitrate staining (Fontana–Masson stain) after direct CAP treatment (60 s) and UV irradiation (UV-B, 60 s) compared to untreated skin samples. Total black pixels were quantified after (**a**) a single treatment with 72 h incubation, (**b**) repeated treatments within 72 h and (**c**) a single treatment and only 24 h incubation. The columns indicate the mean of the individual values ± standard deviation, and the individual values are shown as black dots. Significances were determined by an unpaired *t*-test (**b**,**c**); for (**a**) the Mann–Whitney test was used. (**a**) *n* = 67–71; (**b**) *n* = 17–28; (**c**) *n* = 5–12; the figure in each case corresponds to the number of individual values used to calculate the significances. **: *p* ≤ 0.01, ***: *p* ≤ 0.001.

CAP was applied to full-thickness skin biopsies only once or repeatedly (four times), and biopsies were incubated for 24 h and 72 h, allowing for the formation of melanin in the melanocytes. The respective treatment scheme is shown on top of each graph in Figure 2. UV light exposure served as a control following the same treatment plan.

A single application of CAP had no influence on the melanin content of epidermal melanocytes after 72 h incubation, similarly to UV-B irradiation (Figure 2a). Interestingly, repeated CAP applications during the period of 72 h enhanced the melanin content significantly (Figure 2b). As expected, UV exposure stimulated the melanin content significantly and proved a good responsiveness of the skin samples that were used. When the skin was exposed to CAP or UV-B radiation followed by a short incubation time of 24 h only, the melanin content also increased significantly, indicating an immediate cellular response (Figure 2c).

### 2.2. Morphology and Viability of Cultured Melanocytes after Treatment with CAP in Comparison to UV and IBMX

**Figure 3:**
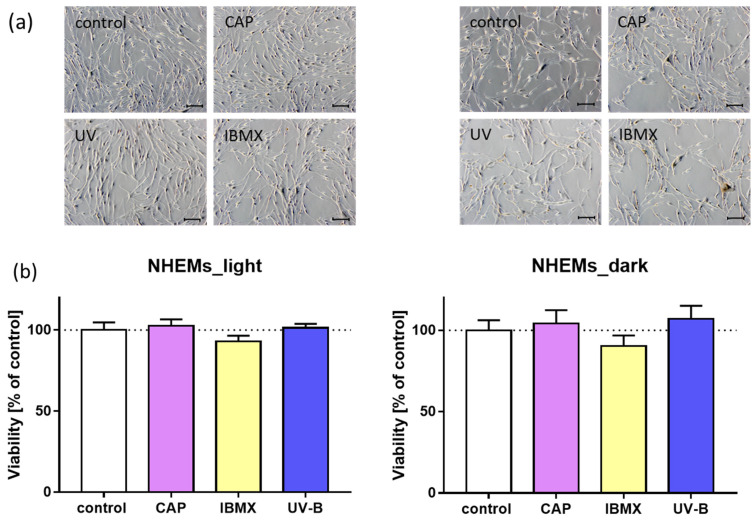
Representative microscopic images of cultured melanocytes and cell viability after treatment with CAP, IBMX and UV-B. (**a**) Light NHEMs (left panel) and dark NHEMs (right panel) maintain their normal cell morphology. Magnification 10×, scale bar 100 µm, phase contrast microscopy. (**b**) Cell viability was assessed by resazurin assay and normalized to the untreated control (*n* = 6). Treatment with CAP, IBMX and UV-B did not affect cell viability.

The impact of CAP on melanogenesis in cultured normal human epidermal melanocytes (NHEMs) was tested in comparison to that of IBMX and UV-B as effective melanin stimulants. Initially, normal cell morphology and the absence of cytotoxic effects were documented. Light microscopy monitoring of cultured cells after treatment revealed no morphological alterations, as shown in representative microscopic images in Figure 3a. Melanocytes formed dendrites and proliferate similarly to the untreated control cells after treatment with CAP, UV and IBMX, respectively. In addition, the cell viability was assessed by a resazurin assay, and untreated cells served as control. The cell viability remained stable after all treatment modalities, as shown in Figure 3b. This was an important prerequisite for carrying out further analysis of the melanin content.

### 2.3. Flow Cytometry for Total Melanin Content in Cultured Human Melanocytes

**Figure 4:**
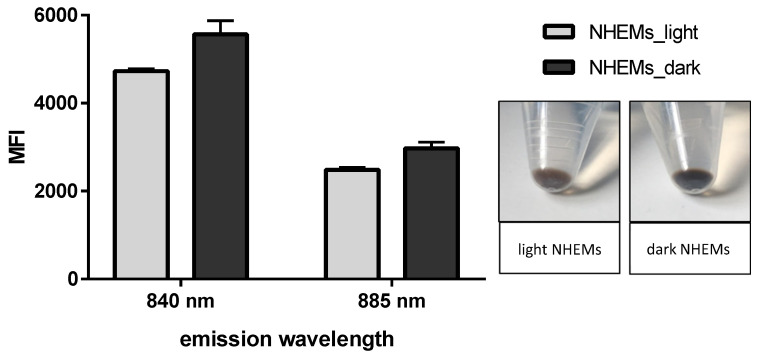
Melanin detection by NIR excitation in light and dark NHEMs. A cell suspension was excited at 808 nm, and emission at 840 nm and 885 nm was detected by flow cytometry. Lighter NHEMs emit a lower fluorescence signal than darker NHEMs at both wavelengths. Bars represent mean fluorescence intensity (MFI) with SD. Insert: Cell pellets of light and dark NHEMs as used for the analysis.

To assess the melanin content on the cellular level, near infrared (NIR) excitation was combined with flow cytometry for analyzing differences in melanin content in a cell population of cultured NHEMs. Initially, cells with different endogenous melanin contents were used to establish the method. As shown in Figure 4, two differently pigmented NHEM cultures were analyzed at emission wavelengths of 840 nm and 885 nm after excitation at 808 nm (NIR), and the mean fluorescence intensity (MFI) was monitored. At both wavelengths, the lightly pigmented cells emitted a lower signal compared to the darker pigmented melanocytes (Figure 4). The mean fluorescence intensities were well correlated with the visual detectable melanin content of these cells. From these findings, it can be concluded that autofluorescence after NIR excitation in combination with flow cytometric analyses is able to detect variations in the melanin content of pigmented cells.

**Figure 5:**
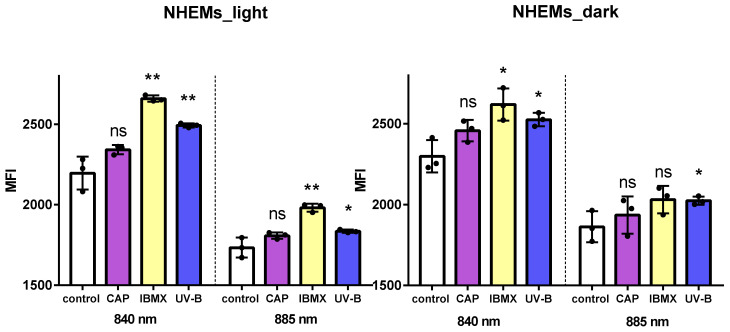
Detection of melanin content by autofluorescence after excitation at 808 nm (NIR) in light and dark melanocytes. NHEMs were analyzed at two emission wavelengths, 840 nm and 885 nm, after treatment with either CAP, IBMX, UV-B or no treatment (control). Increased melanin content results in an increase in MFI. Treatment with IBMX and UV-B revealed higher MFI values than untreated NHEMs. Bars represent mean ± SD. ns: not significant, *: *p* ≤ 0.05, **: *p* ≤ 0.01.

In the next step, cultured NHEMs were challenged by either CAP, IBMX or UV-B treatment and incubated for 72 h. The flow cytometric analysis revealed a significant increase in MFI after treatment with UVB and IBMX in the light and in the dark NHEMs (Figure 5). In both cell types, CAP treatment showed a slightly enhanced MFI after NIR excitation, but the difference was not significant, as summarized in Figure 5. Thus, the total melanin content was considered unaffected in CAP-treated NHEMs.

### 2.4. Pheomelanin and Eumelanin in Cultured Melanocytes after Treatment with CAP, UV and IBMX

Melanocytes contain a mixture of reddish-yellow pheomelanin and brownish-black eumelanin. In fact, the darker melanocytes appear, the higher their eumelanin content. Importantly, the two approaches described above are not suitable for differentiation between melanin types. Here, we have introduced a highly sensitive analytical approach to relatively quantify the two types of melanin in cultured human epidermal melanocytes treated with CAP in comparison to untreated or positive controls (IBMX and UV-B).

The oxidative degradation products of eumelanin and pheomelanin, respectively, result in the formation of specific products. 1*H*-pyrrole-2,4-dicarboxylic acid (PDCA) and 1*H*-pyrrole-2,4,5-tricarboxylic acid (PTCA) serve as marker substances for eumelanin, while 1,3-thiazole-4,5-dicarboxylic acid (TDCA) and 1,3-thiazole-1,4,5-tricarboxylic acid (TTCA) can be assigned to pheomelanin [23,24]. 

**Figure 6:**
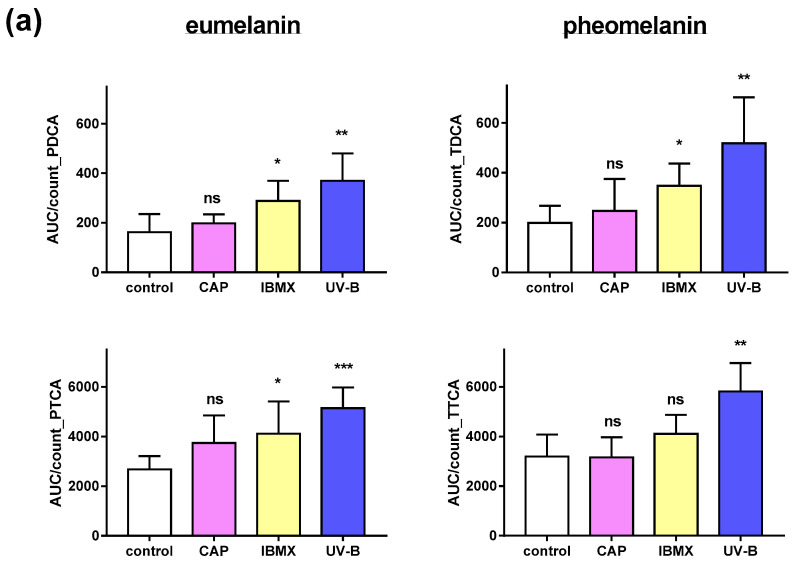

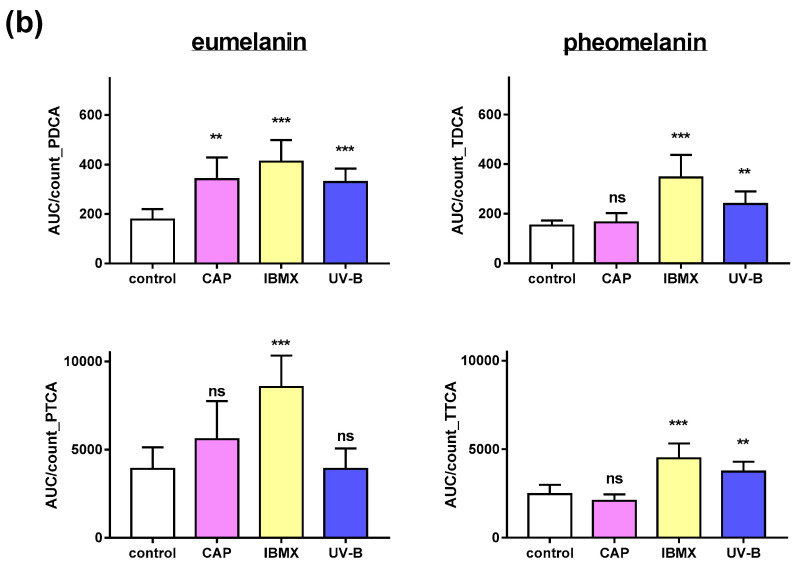
HPLC-MS analysis of eumelanin and pheomelanin in cultured melanocytes. Relative amounts of oxidation products of eumelanin (PDCA and PTCA) and pheomelanin (TDCA and TTCA) in (**a**) lightly pigmented normal human epidermal melanocytes (NHEMs) and (**b**) darkly pigmented NHEMs after treatment with CAP, IBMX and UV-B in comparison to untreated NHEMs (control). The AUC was detected by HPLC-MS using 10^4^ cells in six independent runs and normalized to all cell counts. Statistical analysis was performed using an unpaired *t*-test with *p* ≤ 0.05 (*), *p* ≤ 0.01 (**), *p* ≤ 0.001 (***). ns: not significant.

Following their separation by HPLC, the content of each marker substance was determined by mass spectrometry (MS). This method allows for analysis in a limited sample size of primary cultured melanocytes, i.e., approx. 100,000 cells. Light and dark pigmented NHEMs were included (Figure 6).

As expected, after treatment with UV-B, the amount of eu- and pheomelanin was significantly enhanced, as detected by their degradation products in comparison to their untreated counterpart (Figure 6). This indicates an enhanced melanogenic activity in NHEMs of light- and dark-skinned donor sites. It appeared that darkly pigmented NHEMs responded with an increase only in PDCA but not in PTCA, though both are derived from eumelanin. The phosphodiesterase inhibitor IBMX significantly enhanced the eumelanin content compared to the untreated control cells, and the effect was stronger in darkly pigmented NHEMs. CAP treatment did not have a significant effect on the eumelanin content of the lightly pigmented cells but did reveal significantly more eumelanin-derived PDCA in the darkly pigmented cells (Figure 6b) compared to the untreated control. This result indicates an increase in eumelanin content in the darker NHEMs. The content of pheomelanin remained unchanged in both types of NHEMs after CAP treatment. Melanogenic activity appeared to be inducible, as treatment with UV-B and IBMX resulted in a higher level of eu- and pheomelanin degradation products. This was the case for light and dark melanocytes. In summary, CAP treatment had only a minor effect on melanin synthesis in cultured NHEMs compared to IBMX and UV-B.

## 3. Discussion

As a non-invasive treatment tool, CAP applications in dermatology are widely appreciated, with a substantial number of studies focusing on wound healing, skin cancer and precancerous lesions [14,25,26,27]. In this study, the influence of CAP on melanogenesis in epidermal melanocytes was investigated by detecting the total melanin content with two different approaches and by analyzing the subtypes of melanin. The interventions with CAP in comparison to UV-B radiation were conducted either on full-thickness skin samples or on isolated cultured melanocytes, which additionally received IBMX treatment as a potent enhancer of melanogenesis. Consequently, the methods of detection of melanin were adapted to the treated target structure and included histological and cellular analysis. The results revealed an increased melanin content in CAP-treated and UV-treated skin samples after single and repeated treatment when the incubation time was prolonged to 72 h. The response intensity was similar between both treatment modalities.

In contrast, the flow cytometric approach using NIR excitation based on the autofluorescence of melanin revealed no significant change in melanin content after CAP treatment, while treatment with UV-B and IBMX increased the fluorescence intensity, indicative of a higher melanin content. The analysis of degradation products of melanin, enabling a differentiation between eu- and pheomelanin, indicated a weak increase in eumelanin content after CAP exposure only in darkly pigmented melanocytes but not in pheomelanin content. Possibly, the higher capacity of darkly pigmented melanocytes compared to that of lightly pigmented ones is responsible for the uniqueness of this observation. Interestingly, only the level of PDCA, the degradation product related to 5,6-dihydroxyindole (DHI)-derived eumelanin, was elevated after CAP treatment, while the level of PTCA which is related to 5,6-dihyroxyindole-2-cacboxylic acid (DHICA), was unaffected. This might indicate an effect of CAP on tyrosinase, which is thought to be the enzyme responsible for the polymerization of DHI to form the macromolecule eumelanin, but not on the DHICA oxidase, which is the corresponding enzyme for DHICA-derived eumelanin polymerization [28]. Treatment with UV and IBMX resulted in higher eu- and pheomelanin contents, respectively. Overall, these findings showed a rather small effect of CAP on melanogenesis in full-thickness skin and none in cultured melanocytes compared to UV and IBMX treatment.

The different results between cultured cells and skin samples suggest that the surrounding keratinocytes in human skin may play a role in the activation of melanin-stimulating signal transduction and the secretion of signaling molecules. A direct interaction of CAP with basal melanocytes seems less likely, although the question of skin and generally tissue penetration depth of CAP-generated components is still under debate [29,30]. Partecke et al. impressively showed effects on tumor cells in treated tissue, with up to 50 µm “depth of effective tissue penetration” [31]. Previously, it was shown that epidermal keratinocytes responded with enhanced proliferation in CAP jet-treated skin samples, which may be attributed to bystander effects in combination with cellular signaling cascades [32]. As a natural part of sunlight, UV-B radiation penetrates the epidermis down to the basal layer and can directly modify the activity of melanocytes [4,33,34]. For applications in skin pigmentation disorders, the balance of pro-melanogenic stimuli and anti-melanogenic effects by enhanced epidermal proliferation, for instance, should be considered.

However, the penetration of reactive species generated by CAP is limited, and only stable ROS, if any, may reach deeper layers of the epidermis [29,35]. Short-lived ROS will not directly reach the cells of the basal layer but will interact with the cells of more apical epidermal layers. Epidermal melanogenesis may be influenced by neighboring keratinocytes through paracrine signaling and cytokine secretion [36,37]. Previously, it was reported that CAP application can initiate cellular signaling pathways such as Nrf2 and HIPPO [38,39]. Interestingly, both pathways have been described as important components in melanocytes and their physiological response to oxidative stress and melanogenesis [40,41,42]. However, with respect to melanogenesis, overexpressed Nrf2 may act as a negative regulator [43], and in patients with the depigmentation disorder vitiligo, the pathway is impaired, contributing to melanocyte degeneration [44]. To date, the precise downstream pathways triggered by CAP in melanocytes require further research.

Full-thickness skin samples were exposed directly to the CAP effluent of the plasma jet kINPen^®^ MED, and enhanced melanin synthesis was detected after repeated application and appropriate incubation time. The process of melanin synthesis takes place in several steps at different time intervals, as is well described for UV-B-induced skin tanning [45]. Considering our results, the incubation time after exposure and the intensity seem to be important for CAP-induced melanin synthesis. Repeated CAP treatment of skin samples over a duration of 72 h was still more effective than a single exposure in 72 h. Delayed tanning occurs within days and is due to a well-orchestrated interplay of epidermal keratinocytes and melanocytes, resulting in the accumulation of melanin in the epidermis [4,46,47]. Epidermal keratinocyte signals like p53 and cAMP activate proopiomelanocortin (POMC) expression, which in turn activates microphthalmia-associated transcription factor (MITF) in melanocytes [2,48]. This transcription factor upregulates the expression of the key enzyme tyrosinase and finally stimulates the melanogenic pathway. An enhanced expression of MITF and tyrosine related protein 1 (TRP1) was indeed shown for CAP-treated B16F10 melanoma cells, as well as enhanced cAMP levels and increased melanin content [49]. However, since the malignant form of melanocytes may reveal different reactions compared to healthy primary cells, the effects on primary melanocytes remain to be investigated.

Another factor to consider is the type of CAP device and the operation parameters. In the case of plasma jets, different working gases can be used, and the admixture of oxygen increases the concentration of ROS. In dielectric barrier discharges (DBDs) the ROS concentration very much depends on the ambient conditions, as usually no working gas is supplied.

Flow cytometry is a powerful tool for characterizing functional differences and activation states of whole cells, particularly applicable to living cells and small cell numbers. The use of lasers and photodetectors enables the assessment of fluorescence intensities. The biomolecule melanin comprises distinct biophysical functions; one is the emission of autofluorescence after excitation by near infrared (NIR) radiation. Recently, NIR autofluorescence was reported as an appropriate tool to quantify melanin in different cell lines in vitro [50,51,52]. In this novel approach, the autofluorescence was obtained upon NIR excitation by flow cytometry, enabling the analysis of differences in endogenous or induced melanin content of a small cell population. Moreover, the recovery of viable cells is possible and may offer the option for further cellular analysis. In addition to cellular analysis, Huang et al. proposed that NIR autofluorescence spectroscopy can potentially capture differences in melanin types for pigmented skin lesions in vivo [51].

UV-B irradiation is a well-known inducer of intracellular ROS, which act as a second messenger activator for melanogenesis. Therefore, it was used and applied directly to the cells with a fairly low intensity of 91 mJ/cm^2^, still resulting in the activation of melanin formation, detectable by NIR-induced fluorescence as well as by analysis of specific melanin degradation products. In contrast, IBMX stimulates melanin synthesis by the inhibition of phosphodiesterase, thereby elevating intracellular cAMP levels [53]. The change in melanin content in NHEMs after UV-B irradiation and that after IBMX treatment were very similar, indicating a good melanin synthesizing capacity of both cell types, lightly and darkly pigmented cells. However, in our hands, the melanin content of cultured melanocytes was not significantly affected by CAP, as detected by NIR flow cytometry and HPLC-MS of melanin degradation products. Cultured melanocytes were exposed to CAP by plasma-treated liquids, which is usually less potent than direct exposure, as previously shown for melanoma cell toxicity [54]. Only long-lived ROS such as H_2_O_2_ come in contact with cells and interact with cellular structures. Other components of CAP such as UV radiation and electric fields are missing in this treatment modality, which should be considered a factor.

The synthesis of melanin is the response of melanocytes to protect the human skin against damaging effects of UV radiation. And both types of melanin, eu- and pheomelanin, are positively related to skin color [55,56]. In this context, it is important to note that eumelanin has strong photoprotective properties, while pheomelanin is widely considered a risk factor for skin cancer due to generated ROS after UV radiation [55,57].

In order to differentiate these two types of melanin, the method of Ito et al. was adapted [58], in which oxidative degradation products are analyzed.

Specifically, the melanin is oxidized with hydrogen peroxide in alkaline potassium carbonate solution, resulting in the formation of specific degradation products: PDCA and PTCA serve as marker substances for eumelanin, while TDCA and TTCA originate from pheomelanin [28,58,59]. To adapt this method to the requirement of a small sample volume due to limited material in primary cell culture, mass spectrometry (MS) was coupled with prior separation by HPLC. By this approach, only 100,000 cells were used, which represents an improvement in sensitivity by a factor of 10 in comparison to the published method by Ito et al. [58].

CAP treatment increased the PDCA content in darkly pigmented cells, indicating an increased eumelanin content, but not in lightly pigmented cells, as our results revealed. The pheomelanin content remained unchanged. In contrast, eu- and pheomelanin content was significantly increased after UV and IBMX treatment. Tyrosine and dopaquinone are common precursors for both melanin types [60]. However, pheomelanin is only formed in the presence of cysteine or sulfur-containing compounds [61]. As these readily undergo oxidation when exposed to CAP, they may not be available for pheomelanin formation. The underlying mechanism is not known so far and should be investigated in further studies.

To summarize, we can state that quantifying melanin by feasible means in cultured primary melanocytes is still challenging owing to the small cell numbers and delicate culture conditions. Experimental approaches using full skin biopsies offer a more complex and realistic test system for studying epidermal melanin response.

There are only a few studies available regarding the effect of CAP on melanogenesis in vivo. In a recent study, skin parameters were investigated in vivo after a spark discharge device was applied on rats. Melanin content was significantly increased shortly after intervention and up to 2 weeks later but normalized in the 4 weeks of follow up [62]. Zhai et al. investigated CAP-activated hydrogels for the treatment of the depigmentation disorder vitiligo and achieved partial and complete repigmentation in patients and in a vitiligo-like mouse model [63]. These studies support the beneficial exploitation of CAP for pigmentary applications. However, in our experimental approaches, the melanogenic effects of CAP were inferior to those of well-established stimulants like UV-B and IBMX. Based on these results, the usefulness of CAP to treat skin pigmentation disorders is to be assessed cautiously. However, considering the frequent use of CAP for wound healing and other dermatological applications, the risk of inducing hyperpigmentation as an adverse effect seems to be neglectable.

## 4. Materials and Methods

### 4.1. Chemicals and Reagents

The reference substances 1*H*-pyrrole-2,4-dicarboxylic acid (PDCA) and 1*H*-pyrrole-2,4,5-tricarboxylic acid (PTCA) were purchased from Fluorochem (Hadfield, UK). K_2_CO_3_, Na_2_SO_3_, HCl and H_2_O_2_ (30%) were purchased from Carl Roth GmbH + Co. KG (Karlsruhe, Germany), and resazurin sodium salt was purchased from Alfa Aesar (Haverhill, MA, USA). HCOOH, 3-isobutyl-1-methylxanthine (IBMX) and synthetic melanin were purchased from Sigma-Aldrich Chemie GmbH (Munich, Germany); MeOH and water (MS-grade) were obtained from VWR International (Radnor, PA, USA). All substances were stored at appropriate temperatures and conditions. All solutions were prepared with deionized water unless stated otherwise.

### 4.2. Exposure to CAP and UV

CAP was generated by the atmospheric pressure argon jet kINPen^®^ MED (neoplas med GmbH, Greifswald, Germany). A high electrical voltage at a high frequency of 1 MHz is generated at a stainless-steel electrode, which is centered inside a ceramic capillary attached to a grounded electrode. Intermittent high-frequency discharges at a frequency of 2.5 kHz are used to convert the carrier gas argon into plasma. Argon was used at a constant gas flow rate of 5 standard liters per minute (slm); (AirLiquide, Krefeld, Germany). The generated plasma is carried out of the ceramic capillary by the gas flow and becomes visible as plasma effluent. For skin treatment, the plasma jet was positioned perpendicular to the skin surface at a distance of 12 mm. Cell culture medium was treated with the plasma device for the indicated time and transferred immediately to the cultured cells (see below, indirect treatment). UV radiation (2 × 9 W, Philipps, Amsterdam, The Netherlands) was applied for 60 s, equaling 91 mJ/cm^2^. The emitted wavelength of the UV-B lamps was in the range between 290 and 350 nm.

### 4.3. Skin Samples and Treatment Scheme

Skin samples were derived from routine skin surgery at the clinic of maxillofacial surgery/plastic surgery at the Greifswald University Medical Center and the clinic of dermatology at the Rostock University Medical Center. Ethical approval was provided by the local authorities and registered under BB61/11b and A 2018-0216, respectively. Upon arrival, skin samples were washed in solution containing 10,000 IU penicillin/10,000 µg/mL streptomycin and 250 µg/mL amphotericin B, and the skin was trimmed. Punch biopsies of 5 mm diameter were taken and transferred to serum-free Williams E medium supplemented with 1% l-glutamine. For intervention, skin biopsies were treated either once within 3 days, every 24 h for 3 days, or once within 24 h. The incubation of skin samples took place in Williams E medium. Untreated skin served as control.

### 4.4. Melanin Staining and Image Analyses

Skin samples were snap frozen in liquid nitrogen embedded in Tissue-Tek^®^ O. C. T.™ (Sakura Finetek, Alphen aan den Rijn, The Netherlands), and 6 µm thin sections (minimum 3 per slide) were prepared using a cryotome CM 1950 (Leica, Wetzlar, Germany). Modified Fontana–Masson staining was performed with 10% silver nitrate (AgNO_3_) and ammonia water (28%, until removal of turbidity), followed by incubation for 60 min at 60 °C. Confocal laser scanning microscopy (Operetta CLS, PerkinElmer, Waltham, MA, USA) was employed for capturing images. Image analysis was performed with Fiji software version 1.52t (open source). Several regions of interest (ROIs) of identical size were placed inside each epidermal section while keeping the melanin-containing region centered.

### 4.5. Cell Culture and Treatment Scheme

Normal human epidermal melanocytes (NHEMs) derived from juvenile foreskin of donors with light and dark skin pigmentation, respectively, were purchased from PromoCell (Heidelberg, Germany). Cells were maintained in melanocyte-specific cell culture media CnT-40 (CellnTec, Bern, Switzerland) at 37 °C and 5% CO_2_ and were passaged approximately once a week.

For experimental intervention, cells of passage 1–6 were seeded in 12-well cell culture plates at a density of 100,000 cells/well and allowed to attach for 24 h. For treatment with CAP, 5.5 mL of cell culture medium was exposed to the plasma effluent of the kINPen^®^ MED at a distance of 13 mm for 60 s. Directly after treatment, 1 mL plasma-treated cell culture medium was transferred to the adherent cells. For UV radiation, the supernatant was removed, and the cells were exposed to UV-B radiation. For IBMX treatment, a stock solution of 10 mmol/L was prepared by dissolving 2.25 mg IBMX in 1 mL DMSO (Sigma-Aldrich, München, Germany). Then, 10 µL of the stock solution was diluted to 100 µmol/L in cell culture medium and transferred to the cells. Untreated cells served as the control.

### 4.6. Cell Viability and Cell Count

For monitoring the cell viability after intervention, a resazurin assay was performed. In a 12-well cell culture plate, the resazurin reagent (100 µmol/L) was added to adherent cells and incubated for 2 h. The supernatant was transferred to a 96-well-plate, and fluorescence intensity was read at 590 nm after excitation at 530 nm (Tecan^®^, Maennedorf, Switzerland).

The cell number was determined prior to each experiment using an Attune^®^ flow cytometer (Thermo Fisher Scientific, Waltham, MA, USA). Additionally, the number of total counts of each probe was evaluated after the end of incubation time.

### 4.7. Flow Cytometry

In order to detect autofluorescence upon NIR excitation in NHEMs, a Cytoflex LX flow cytometer (Beckman Coulter, Brea, CA, USA) with laser excitation at 808 nm was used. The emitted autofluorescence was detected at 840 nm and 885 nm, respectively, in resuspended vital cells.

### 4.8. Sample Preparation and Melanin Degradation

Degradation was performed as described earlier by Ito et al. and slightly modified [58,64]. After the indicated incubation time, cells were detached using accutase (BioLegend, San Diego, CA, USA), and the number of total events in each sample was determined. The cells were washed with deionized water, and the cell pellet was lyophilized (Martin Christ Gefriertrocknungsanlagen GmbH, Osterode am Harz, Germany). The lyophilized cells were resuspended with 25 µL H_2_O, and alkaline hydrogen peroxide oxidation was conducted by adding 6.25 µL H_2_O_2_ 30% and 93.8 µL 1 mol/L K_2_CO_3_. Samples were incubated for 20 h at room temperature (21 ± 2 °C) in a shaker at 750 rpm. The remaining hydrogen peroxide was decomposed by adding 12.5 µL Na_2_SO_3_ (10%), and the samples were acidified with 35 µL 6 mol/L HCl. Finally, samples were filtered through a 0.2 µm regenerated cellulose syringe filters (Macherey-Nagel, Düren, Germany). 

### 4.9. LC-MS Conditions

The LC-MS-system (Shimadzu, Kyoto, Japan) consisted of a CBM-20A controller unit, pumps (LC-20AD), a SIL-20A_HT_ auto sampler, a CTO-10AS column oven, a valve unit FCV-20AH_2_ and an MS-8030. The mobile phase was 0.2% HCOOH. MeOH was added in the following gradient: 0–3.0 min 1% MeOH, 3.1–6.0 min 15% MeOH, 6.1–10.0 min 1% MeOH. The flow rate was set to 1 mL/min. A C18 column (Kinetex 4.6 × 100 mm 2.6 µm 100 Å) was kept at 50 °C. Samples (10 µL) were auto-injected. 

For MS detection, electrospray ionization (ESI) was applied as the ion source, while the combination of a triple-quadrupole and an electron multiplier were used as analyzer and detector. Detection times were set from 3.0 min to 10.0 min. 

PTCA and TTCA were measured using the positive selected ion monitoring mode in the third quadrupole (Q3-SIM), while PDCA and TDCA were measured using the negative Q3-SIM mode. PDCA and PTCA were identified both by reference substances and mass-to-charge ratio (*m*/*z*) (PDCA 154.0; PTCA 200.0). TDCA and TTCA were identified by mass-to-charge ratio (TDCA 172.0; TTCA 218.0) only. The latter were not commercially available. The area under the curve (AUC) obtained by MS analysis was normalized to the total count of all events in the range of 0.5 µm to 50 µm (see Section 4.6). The AUC per count (AUC/count) was used to compare relative amounts of specific degradation products.

### 4.10. Statistics

GraphPad Prism software (version 7.00) was used to calculate and create the diagrams. Unless otherwise stated, the arithmetic mean value with the correspondingly calculated standard deviation (SD) is given. Unless otherwise stated, significance was calculated using an unpaired *t*-test (*p* ≤ 0.05) after previous testing for normal distribution using a Shapiro–Wilk test. The significances were determined according to their empirical significance level, indicated as following: ns: not significant; *: *p* ≤ 0.05; **: *p* ≤ 0.01; ***: *p* ≤ 0.001.

## 5. Conclusions

In conclusion, the melanin content of cultured NHEMs remained unchanged after CAP treatment, as shown by NIR autofluorescence and HPLC-MS of melanin degradation products. Both positive stimulants, UV and IBMX, revealed the expected increase in melanin content and proved the responsiveness of the cells. Melanocytes inside whole-skin biopsies showed increased melanin deposition after CAP treatment, which indicates a role for neighboring epidermal keratinocytes and melanogenic signaling.

The risk of hyperpigmentation as a result of CAP application during wound healing can be considered marginal.

## Data Availability

The data that support the findings of this study are available on request from the corresponding author, [S.H.].
